# Death-associated protein kinase 1 mediates interleukin-1β production through regulating inlfammasome activation in Bv2 microglial cells and mice

**DOI:** 10.1038/s41598-018-27842-y

**Published:** 2018-07-02

**Authors:** Limin Song, Lei Pei, Lisha Hu, Shangwen Pan, Wei Xiong, Min Liu, Yan Wu, You Shang, Shanglong Yao

**Affiliations:** 1 Department of Anesthesiology, Union Hospital, Tongji Medical College, Huazhong University of Science and Technology, Wuhan, 430022 China; 2 Department of Neurobiology, Tongji Medical Collge, Huazhong University of Science and Technology, Wuhan, 430022 China; 3 Department of Critical Care Medicine, Union Hospital, Tongji Medical College, Huazhong University of Science and Technology, Wuhan, 430022 China; 4 Department of Neurology, Union Hospital, Tongji Medical College, Huazhong University of Science and Technology, Wuhan, 430022 China; 5 Institute of Anesthesiology and Critical Care Medicine, Union Hospital, Tongji Medical College, Huazhong University of Science and Technology, Wuhan, 430022 China

## Abstract

Interleukin-1β (IL-1β) plays a crucial role in mediating inflammation and innate immunity response in the central nervous system. Death-associated protein kinase 1 (DAPK1) was shown to be involved in several cellular processes. Here, we investigated the effects of DAPK1 on IL-1β production in microglial cells. We used a combination of *in vitro* (Bv2 microglial cell cultures) and *in vivo* (mice injected with amyloid-β (Aβ)) techniques to address the role of caspase-1 activation in release of IL-1β. DAPK1 involvement was postulated through genetic approaches and pharmacological blockade of this enzyme. We found that Aβ_25–35_ stimulation induced IL-1β production and caspase-1 activation in LPS-primed Bv2 cells and mice. DAPK1 knockdown and catalytic activity inhibition reduced IL-1β maturation and caspase-1 activation, nevertheless, DAPK1 overexpression attenuated these effects. Aβ_25–35_-induced lysosomal cathepsin B leakage was required for DAPK1 activation. Furthermore, repeated DAPK1 inhibitor treatment ameliorated the memory impairment in Aβ_25–35_-injected mice. Taken together, our findings suggest that DAPK1 facilitates Aβ_25–35_-induced IL-1β production through regulating caspase-1 activation in microglial cells.

## Introduction

Death-associated protein kinase 1 (DAPK1) is a member of death domain-containing calcium/calmodulin-dependent serine/threonine kinase family that plays a critical role in regulating several cellular processes including cell apoptosis and autophagy^1–4^. The activity of DAPK1 is regulated through its Ser^308^ residue located within its auto-regulatory domain^5^. Recent studies have implied the involvement of DAPK1 in inflammatory signals and innate immunity of different biological systems^2^.

Alzheimer’s disease (AD), a prevalent neurodegenerative disorder that is characterized by extensive extracellular deposits of the neurotoxic β-amyloid (Aβ) in senile plaques, and neuronal intracellular accumulation of neurofibrillary tangles formed by tau proteins^6,7^. Aβ deposition recruits and activates microglia, which results in the production of various pro-inflammatory mediators that ultimately lead to neuronal injury^8–12^. Interleukin-1β (IL-1β), which is massively accumulated in the brains of individuals with AD, has been certified as a central driving force in the neuroinflammation during AD development^13,14^.

IL-1β is synthesized as the inactive precursor pro-IL-1β via the nuclear factor-κB (NF-κB) pathway and then processed into its mature form by caspase-1 which itself is tightly controlled by intracellular inflammasomes^15^. The NOD-, LRR- and pyrin domain-containing 3 (NLRP3) inflammasome, which consists of NLRP3, the adaptor protein apoptosis-associated speck-like protein that contains a caspase activating recruitment domain (ASC), and caspase-1, is the most intensively studied inflammasome complex, since it has been implicated to sense multiple pathogen-associated molecular patterns and endogenous danger signals^16^. Generally, activation of the NLRP3 inflammasome requires two steps. The first step is priming through up-regulating the expression of NLRP3 via the NF-κB pathway^17^; the second step is exposing to the following or simultaneous NLRP3-specific activators that induce the assembly of the inflammasome, which leads to the autocatalytic activation of caspase-1^18^. It has been shown that particulate stimuli lead to the leakage of lysosomal cathepsin B into the cytosol, where this protease binds to NLRP3 and activates it^19–21^.

Here, we carried out a set of *in vitro* and *in vivo* experiments to investigate the role of DAPK1 in IL-1β production in microglia and the possible molecular mechanisms. Our findings demonstrate that DAPK1, which is activated in the lysosomal protease cathepsin B-dependent pathway, functions as a positive regulator of the inflammasome activation. Moreover, DAPK1 inhibitor administration reduces IL-1β production through inhibiting NLRP3 inflammasome activation and improves cognitive outcomes in Aβ_25–35_-injected mice.

## Results

### Aβ_25–35_ induces IL-1β production and DAPK1 activation in LPS-primed Bv2 cells

**Figure 1:**
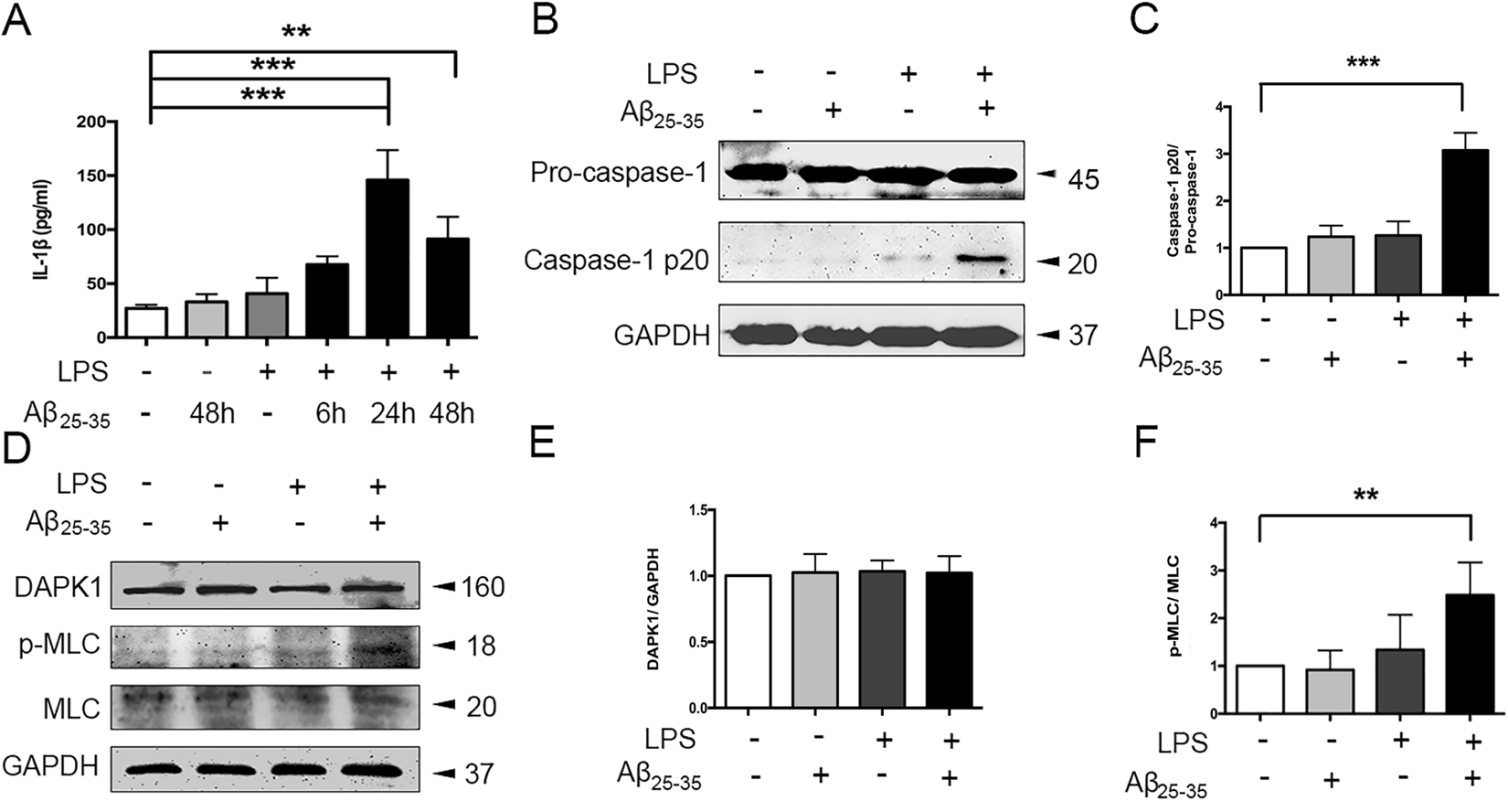
Aβ_25–35_ induced caspase-1 and DAPK1 activation in LPS-primed Bv2 cells. (**A**) Cells were primed with LPS (100 ng/ml) for 6 h, and treated with Aβ_25–35_ (25 μM) for varying times (6 h, 24 h, 48 h); the amount of IL-1β in the culture supernatant was assessed by ELISA. (**B**–**F**) Cells were primed with LPS and stimulated with Aβ_25–35_ for 24 h. Protein levels of caspase-1 (**B–C**), DAPK1 and p-MLC (**D–F**), see original blots in Supplementary Fig. S2) were assessed by western blotting analysis. Data are shown as mean ± SEM for three independent experiments. **P < 0.01, ***P < 0.001. Aβ: β-amyloid; DAPK1: death-associated protein kinase 1; LPS: lipopolysaccharide; MLC: myosin II regulatory light chain.

We first examined the effect of amyloid-β (Aβ) on IL-1β production in Bv2 cells. Considering that pro-IL-1β is not constitutively expressed in microglia, we transiently activated the cells with LPS (100 ng/ml) for 6 h to induce robust pro-IL-1β transcription^22^. As shown in Fig. 1A, IL-1β concentration in the culture supernatant began to increase at 6 h, peaked at 24 h, and maintained at 48 h after Aβ_25–35_ (25 μM) treatment in LPS-primed cells. No substantial increase of IL-1β levels was detected after treatment with both fibrillar and oligomeric preparations of Aβ_1–42_ in LPS-primed cells (see Supplementary Fig. S1). Furthermore, Aβ_25–35_ treatment for 24 h induced a significant increase in the cleavage of caspase-1 in LPS-primed Bv2 cells (Fig. 1B-C).

Next, we sought to investigate whether Aβ_25–35_ had an effect on DAPK1 activity. Since DAPK1 phosphorylates myosin II regulatory light chain (MLC) immediately upon activation, we measured the expression of phosphorylated MLC (p-MLC) at the protein levels as the indicator of DAPK1 activity^23^. Although total DAPK1 levels were comparable among all the groups, the protein levels of p-MLC were significantly increased in LPS-primed cells following Aβ_25–35_ treatment (Fig. 1D–F), indicating an increase in DAPK1 activity.

### Cathepsin B leakage is required for Aβ_25–35_-induced IL-1β production and caspase-1 activation in LPS-primed Bv2 cells

**Figure 2:**
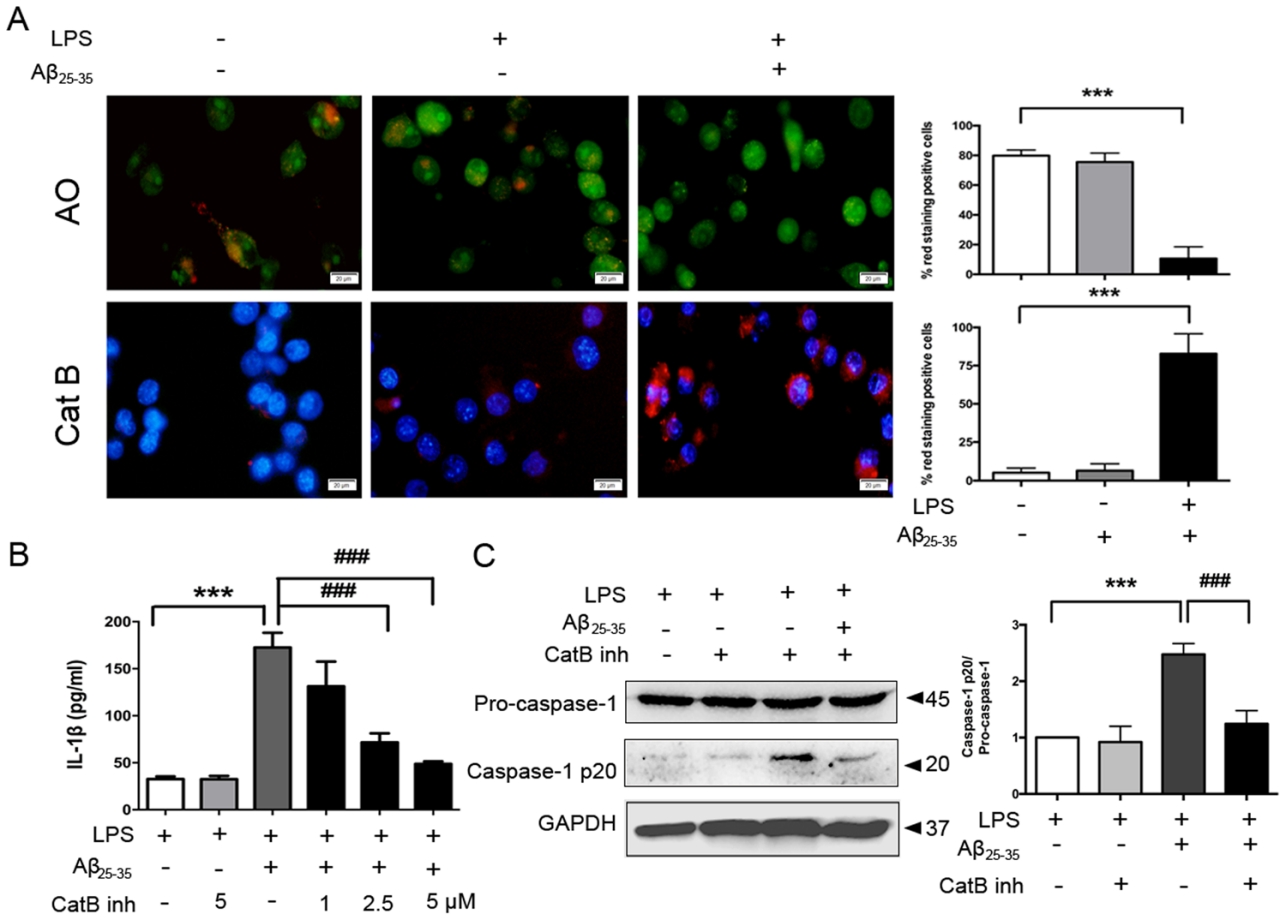
Aβ_25–35_-induced IL-1β production in LPS-primed Bv2 cells is dependent on cathepsin B. (**A**) Fluorescent images of acridine orange (AO) and a cathepsin B substrate in cells unprimed or primed with LPS (100 ng/ml) alone or primed with LPS plus Aβ_25–35_ (25 μM) stimulation were exemplified (12 images per condition). Scale bar = 20 μm. (**B**) LPS-primed cells were treated with different doses (1, 2.5, 5 μM) of cathepsin B inhibitor for 1 h before Aβ_25–35_ exposure. Amounts of IL-1β in the culture medium after 24 h of Aβ_25–35_ treatment were measured by ELISA. (**C**) LPS-primed Bv2 cells were treated with cathepsin B inhibitor (5 μM) for 1 h before Aβ_25–35_ treatment. Protein expression of caspase-1 was examined by western blotting and quantified by densitometry. Data are shown as mean ± SEM for at least three independent experiments. ^***^P < 0.001 and ^###^P < 0.001. AO: acridine orange; Cat B: cathepsin B substrate; Cat B inh: cathepsin B inhibitor.

To directly investigate the changes in lysosome membrane permeability in live microglia, we first stained cells with the acidity-dependent acridine orange (AO), which accumulates in acidic compartments such as intact lysosomes with red fluorescence and displays green staining in the less acidic environment including the cytoplasm and nucleus. The disruption of lysosomal integrity is visualized by decreased red staining and increased green fluorescence^24^. As shown in Fig. 2A, non-primed cells and LPS-primed cells contained red puncta signals for AO, whereas Aβ_25–35_ treatment turned fluorescent signals largely green. We also stained cells with a cell-permeable, fluorescent cathepsin B substrate. As expected, we observed a marked increase of activated cathepsin B leakage into the cytoplasm after Aβ_25–35_ exposure in LPS-primed cells (Fig. 2A).

Next, we treated cells with CA-074Me, a specific inhibitor of cathepsin B. As shown in Fig. 2B-C, CA-074Me significantly lowered Aβ_25–35_-induced IL-1β production in a dose-dependent manner and blocked cleavage of caspase-1 in LPS-primed Bv2 cells.

### DAPK1 is required for Aβ25–35-induced IL-1β production and caspase-1 activation in LPS-primed Bv2 cells

**Figure 3:**
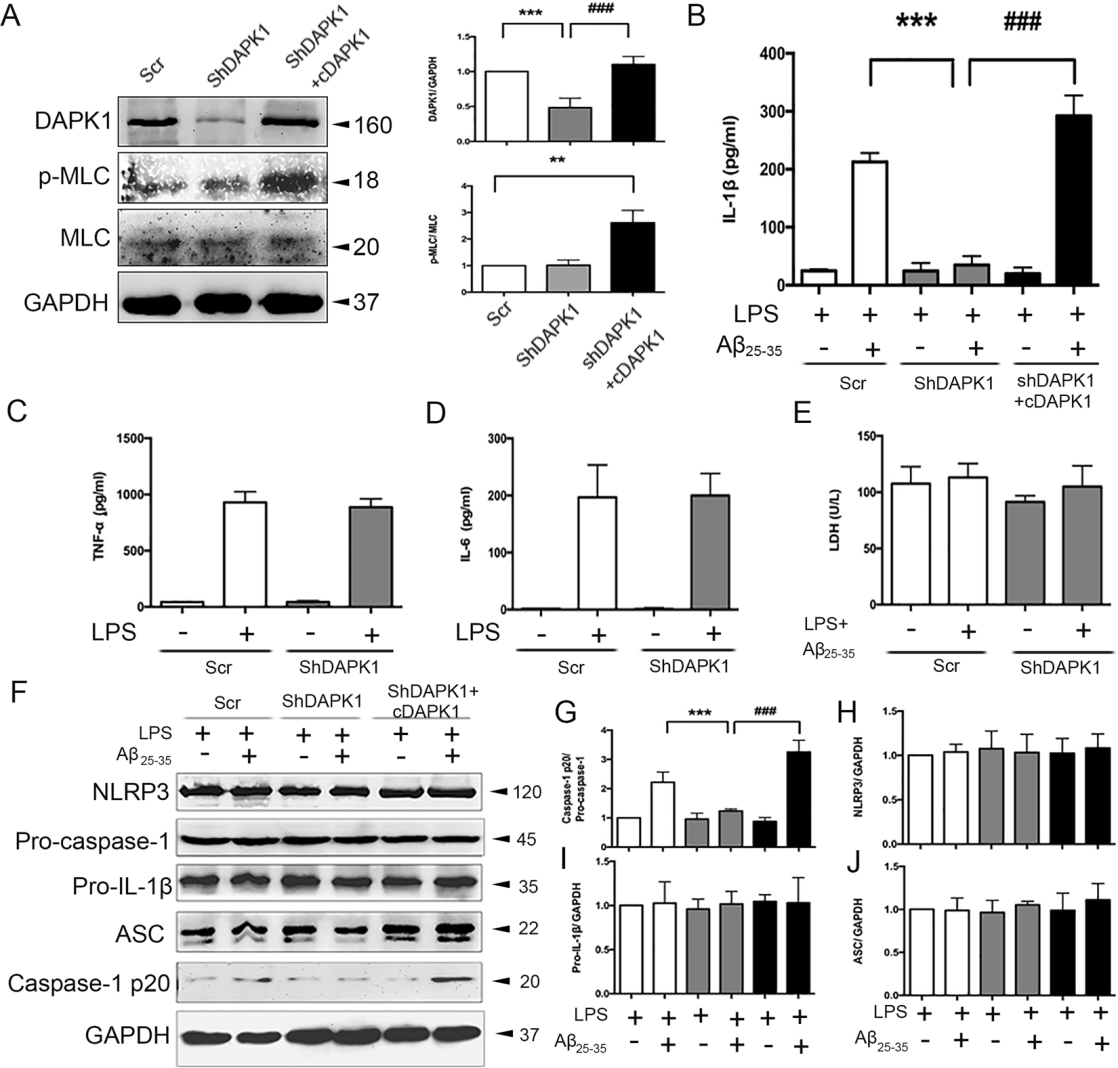
DAPK1 is involved in Aβ_25–35_-induced IL-1β maturation and caspase-1 activation in LPS-primed Bv2 cells. (**A**) Confirmation of DAPK1 knockdown and cDAPK1 expression in Bv2 cells by western blotting analysis (see original blots in Supplementary Fig. S3A). (**B**) The effects of DAPK1 knockdown and cDAPK1 expression on Aβ_25–35_-induced IL-1β secretion were measured by ELISA. (**C–E**) The effects of DAPK1 knockdown on LPS-induced TNF-α and IL-6 secretion, as well as LPS + Aβ_25–35_-induced LDH release were determined. (**F–J**) The effects of DAPK1 knockdown and cDAPK1 expression on the expression of caspase-1, pro-IL-1β, NLRP3 and ASC in LPS-primed cells were determined by western blotting analysis (see original blots in Supplementary Fig. S3B). Results are mean ± SEM for at least three independent experiments. ^**^P < 0.01, ^***^P < 0.001; ^###^P < 0.001. cDAPK1: constructively activated DAPK1 construct; Scr: scrambled shRNA; ShDAPK1: DAPK1-specific shRNA.

To identify the role of DAPK1 in the regulation of IL-β production and NLRP3 inflammasome activation in Bv2 cells, an shRNA-mediated DAPK1 deletion experiment and DAPK1^ΔCaM^, a constitutively active form of truncated DAPK1 construct (here referred to as cDAPK1), expression experiment was performed. As shown in Fig. 3A, DAPK1 expression was dramatically decreased in DAPK1 knockdown cells, and significantly increased in cDAPK1-expressing cells. As expected, p-MLC levels were remarkably up-regulated in cDAPK1-expressing cells.

Simultaneously, we observed that Aβ_25–35_-induced secretion of IL-1β and cleavage of caspase-1 was significantly reduced in DAPK1 knockdown cells and increased in cDAPK1-expressing cells (Fig. 3B,F,G). As a control, the secretion of other pro-inflammatory cytokines, tumor necrosis factor-α (TNF-α) and IL-6, was nearly identical between control and DAPK1 knockdown cells activated by LPS (Fig. 3C-D). Lactate dehydrogenase (LDH) assays were used to evaluate cell viability, and the results showed that DAPK1 knockdown did not affect LDH release, either in the presence or absence of stimulation (Fig. 3E). Notably, the levels of NLRP3 components (NLRP3, ASC, pro-caspase-1) and pro-IL-1β were comparable among all groups (Fig. 3F,H–J).

### Inhibition of DAPK1 catalytic activity attenuates Aβ_25–35_-induced caspase-1 activation in LPS-primed Bv2 cells

**Figure 4:**
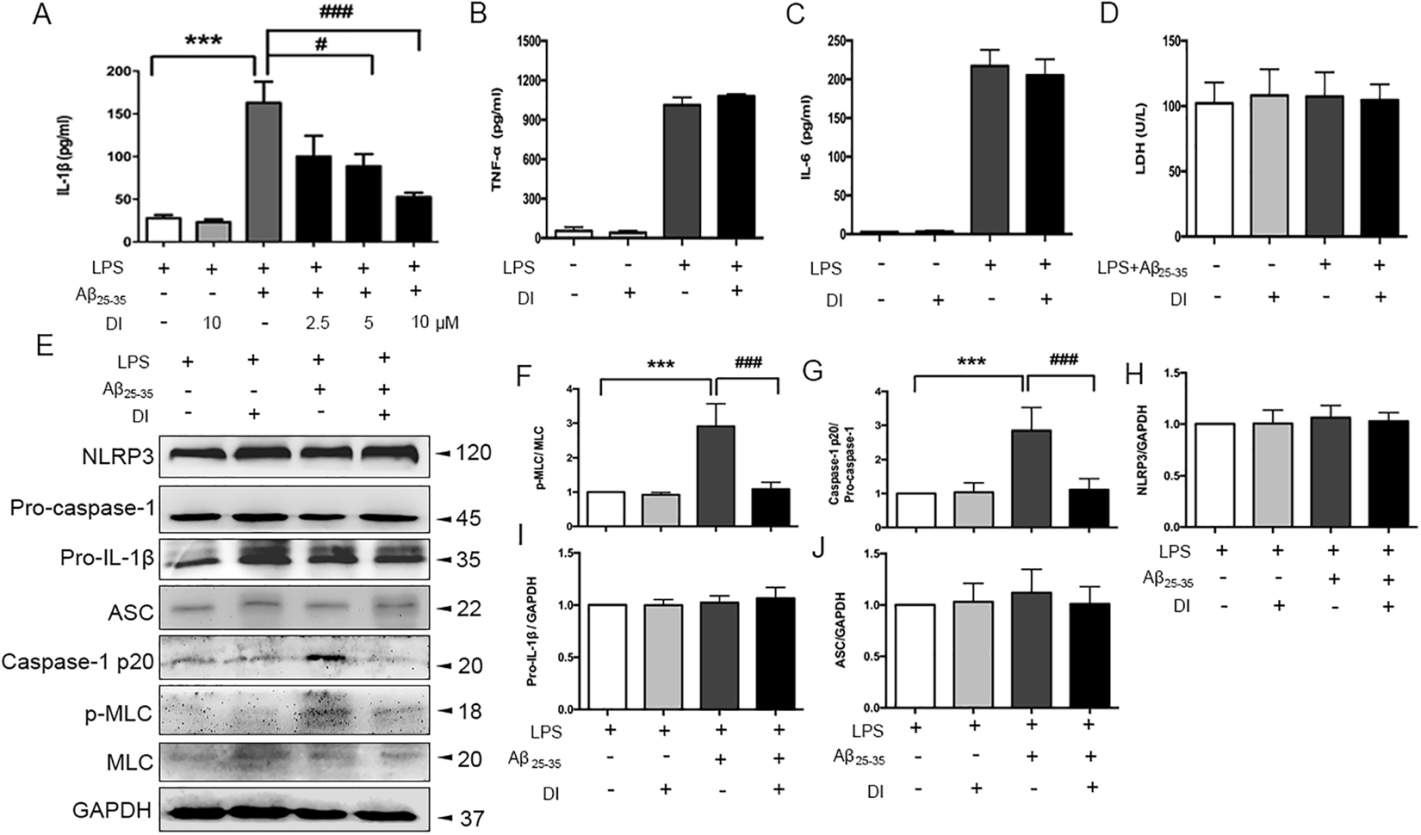
Lack of DAPK1 activity impairs Aβ_25–35_-induced IL-1β maturation and caspase-1 activation in LPS-primed Bv2 cells. (**A**) LPS-primed cells were treated with DAPK1 inhibitor at different doses (2.5, 5, 10 μM) for 1 h before Aβ_25–35_ (25 μM) exposure. Amounts of IL-1β in the culture supernatant after 24 h of Aβ_25–35_ treatment were assessed by ELISA. (**B–D**) The effects of DAPK1 inhibitor (10 μM) treatment on LPS-induced TNF-α and IL-6 secretion, as well as LPS + Aβ_25–35_-induced LDH release were determined. (**E–J**) The effects of DAPK1 inhibitor (10 μM) treatment on the expression of p-MLC, caspase-1, NLRP3, pro-IL-1β and ASC in LPS-primed cells were determined by western blotting analysis (see original blots in Supplementary Fig. S4). Data are expressed as mean ± SEM for at least three independent experiments. ^***^P < 0.001; ^#^P < 0.05, ^###^P < 0.001. DI: DAPK1 inhibitor.

Given that DAPK1 was indispensable for the activation of the NLRP3 inflammasome, the next question was whether the modulation of DAPK1 activity had an influence on NLRP3 inflammasome activation and IL-1β production induced by Aβ_25–35_. LPS-primed Bv2 cells were treated with Aβ_25–35_ with or without pretreatment with a highly selective DAPK1 kinase inhibitor^25^. The inhibition of DAPK1 catalytic activity was evidenced by the decrease in protein levels of Aβ_25–35_-induced p-MLC (Fig. 4E,F). Importantly, we found that DAPK1 inhibitor dose-dependently blunted IL-1β secretion induced by Aβ_25–35_ (Fig. 4A). Furthermore, the inhibition of DAPK1 significantly reduced caspase-1 cleavage (Fig. 4E,G) yet the levels of NLRP3, pro-caspase-1, pro-IL-1β as well as ASC, were nearly identical in all groups (Fig. 4E,H–J). Notably, DAPK1 had no effect on LPS-induced TNF-α and IL-6 secretion, as well as LPS + Aβ_25–35_-induced LDH release (Fig. 4B–D).

### DAPK1 activation is dependent on cathepsin B

**Figure 5:**
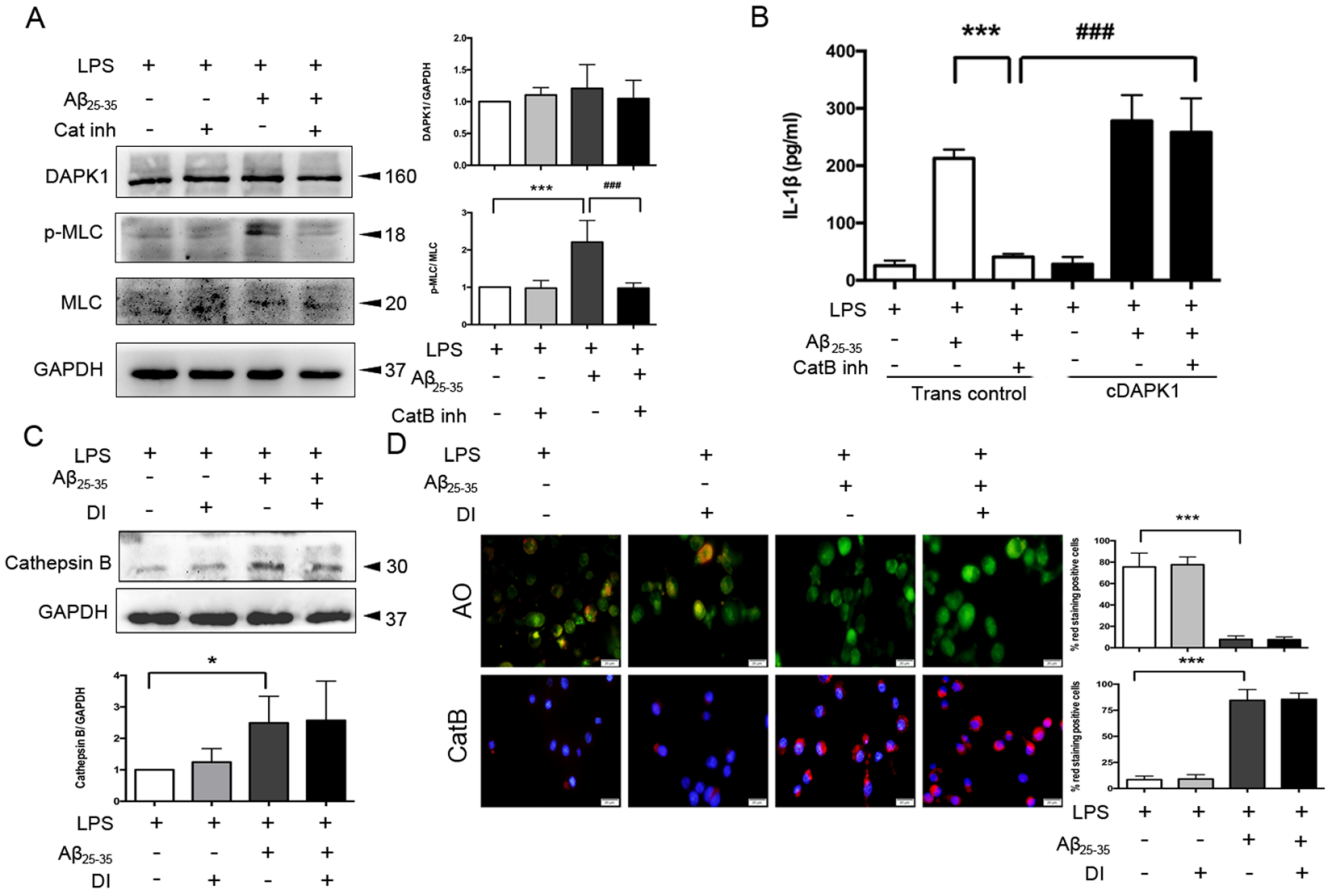
Aβ_25–35_-induced cathepsin B acts upstream of DAPK1. (**A**) The effects of cathepsin B inhibitor (5 μM) treatment on the expression of DAPK1 and p-MLC in LPS-primed, Aβ_25–35_-stimulated Bv2 cells were analyzed by western blotting analysis (see original blots in Supplementary Fig. S5A). (**B**) Cells were transiently transfected with DAPK1 overexpression plasmids (cDAPK1) or empty plasmids (Trans control) using Lipofectamine 2000. The effect of cathepsin B inhibitor (5 μM) treatment on Aβ_25–35_-induced IL-1β secretion in DAPK1 overexpression cells were analyzed by ELISA. (**C–D**) LPS-primed Bv2 cells were treated with or without DAPK1 inhibitor (10 μM), followed by Aβ_25–35_ exposure for 24 h. (**C**) The expression of cathepsin B in the cytoplasm was determined by western blotting analysis (see original blots in Supplementary Fig. S5B). (**D**) Fluorescent images of acridine orange and a cathepsin B substrate were exemplified (12 images per condition). Scale bar = 20 μm. Data are shown as mean ± SEM for at least three independent experiments. ^*^P < 0.05 and ^***^P < 0.001; ^###^P < 0.001. AO: acridine orange; Cat B: cathepsin B substrate; Cat B inh: cathepsin B inhibitor; cDAPK1: constructively activated DAPK1 (DAPK1^ΔCaM^ mutant); DI: DAPK1 inhibitor; Trans con: transfection control.

Considering that DAPK1 interacts with cathepsin B^26^, of further interest was whether cathepsin B mediated the process of DAPK1-involved caspase-1 activation. To investigate whether DAPK1 activation is dependent on cathepsin B release, the effect of CA-074Me on DAPK1 activity was tested in LPS-primed cells. The results showed that CA-074Me treatment had no effect on DAPK1 expression, but abolished the increase in the protein levels of p-MLC induced by Aβ_25–35_ (Fig. 5A), which is indicative of less DAPK1 activation. In addition, overexpression of DAPK1 restored Aβ_25–35_-induced IL-1β release in presence of CA-074Me in LPS-primed cells (Fig. 5B). On the contrary, DAPK1 inhibitor treatment had no influence on the expression (Fig. 5C) and leakage of cathepsin B (Fig. 5D).

### Acute DAPK1 inhibitor treatment attenuates Aβ_25–35_-induced IL-1β production in mice

**Figure 6:**
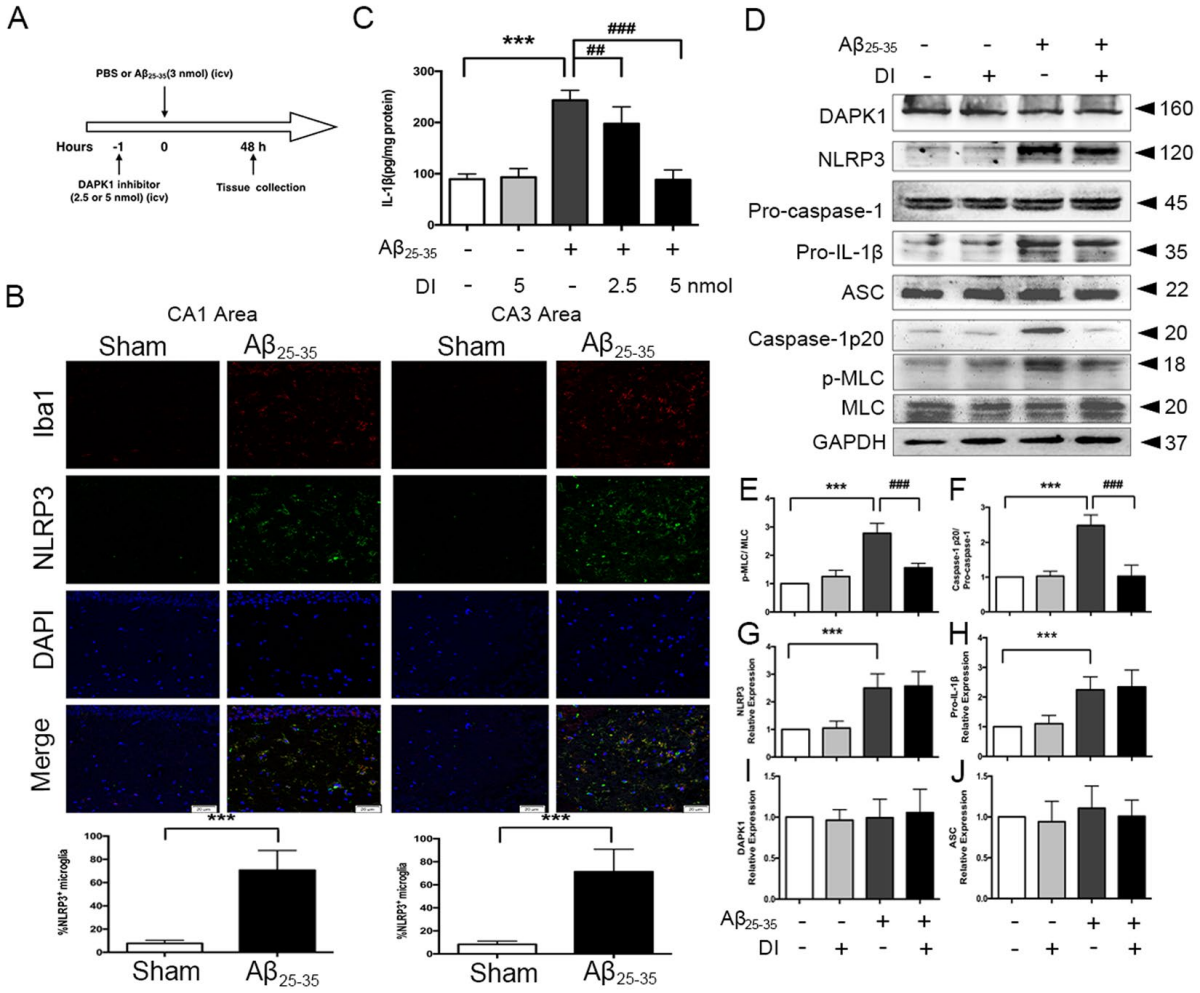
DAPK1 is required for Aβ_25–35_-induced IL-1β production *in vivo*. (**A**) Experimental procedure for neurochemical analysis with acute DAPK1 inhibitor (DI) treatment. (**B**) Co-immunofluorescence of NLRP3^+^ cells co-localized with Iba1^+^ cells in hippocampal CA1 and CA3 regions of Aβ_25–35_-injected mice. Representative images of CA1 and CA3 at X 200 magnification are shown (n = 5, 4 images per animal, 2 images for CA1 area and 2 images for CA3 area). Scale = 50 μm. (**C**) Effects of DAPK1 inhibitor treatment on Aβ_25–35_-induced IL-1β generation in the hippocampus were assayed by ELISA (n = 6). (**D–J**) The expression of DAPK1, p-MLC, NLRP3, caspase-1, pro-IL-1β and ASC in the hippocampus was determined by western blotting analysis (n = 6) (see original blots in Supplementary Fig. S6). Data represent mean ± SEM. ^***^P < 0.001; ^##^P < 0.01, ^###^P < 0.001. DI: DAPK1 inhibitor.

To further test whether the essential role of DAPK1 in caspase-1/IL-1β signaling was also observable *in vivo*, an Aβ_25–35_ injection rodent model was adopted. The experimental scheme of animal treatment and neurochemical analyses is explained in Fig. 6A. Increased microglia marker Iba1-/NLRP3-positive cells were observed in the CA1 and CA3 areas of hippocampus from Aβ_25–35_-injected mice (Fig. 6B). As shown in Fig. 6C-D, compared with the sham + vehicle group, Aβ_25–35 + _vehicle group exhibited increased levels of IL-1β, NLRP3, pro-IL-1β, caspase-1 p20 and p-MLC in the hippocampus. DAPK1 inhibitor was effective in attenuating IL-1β production and caspase-1 cleavage (Fig. 6C,D,F). As expected, this was associated with a decrease in p-MLC protein levels (Fig. 6D,E). The expression of NLRP3 and pro-IL-1β exhibited no difference between the Aβ_25–35 + _vehicle group and Aβ_25–35 + _DAPK1 inhibitor group (Fig. 6D,G,H).

### Subchronic DAPK1 inhibitor treatment improves Aβ_25–35_-induced memory deficits in mice

**Figure 7:**
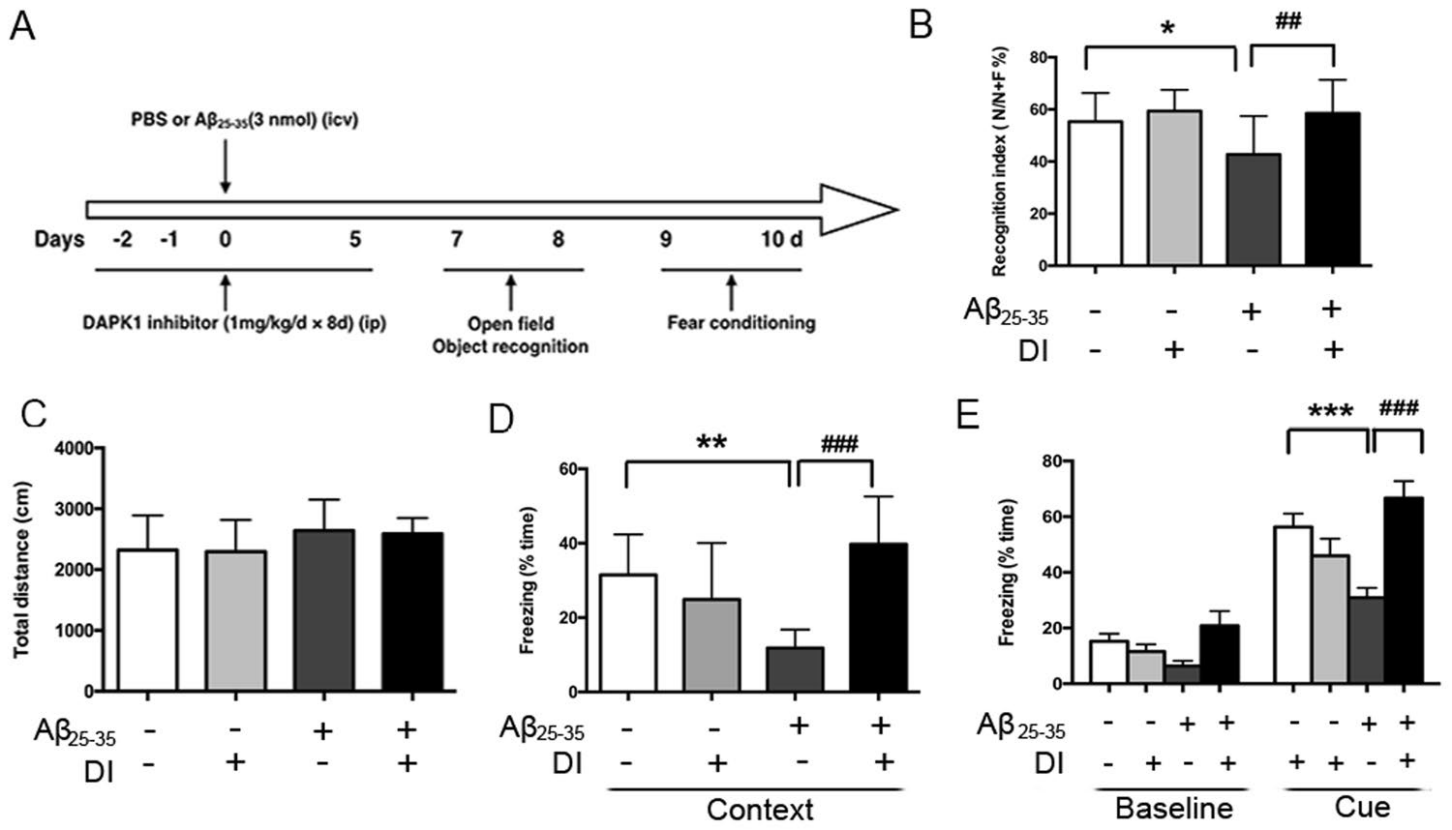
Repeated DAPK1 inhibitor treatment ameliorates Aβ_25–35_-induced memory deficits. (**A**) Time chart of experimental procedure. (**B**) The recognition index during the retention memory phase in the ORT. (**C**) Total distances travelled in the open-field for 5 min were recorded. (**D–E**) Contextual- (**D**) and cue-fear conditioning (**E**) learning of mice, as presented by the percentage freezing time during the tests. Data are presented as mean ± SEM (n = 11). ^*^P < 0.05, ^**^P < 0.01; ^##^P < 0.01, ^###^P < 0.001. Context: contextual-conditioning fear test; Cue: cue-conditioning fear test; DI: DAPK1 inhibitor.

The time chart of animal treatment and behavioral assessments is explained in Fig. 7A. Compared with the sham mice, Aβ_25–35_ injection decreased the recognition index (Fig. 7B), thereby implying a cognitive impairment. Subchronic DAPK1 inhibitor treatment significantly increased the recognition index in Aβ_25–35_-injected mice. Furthermore, it is important to emphasize that the deficit in the ORT observed in Aβ_25–35_-injected mice cannot be explained by a reduced locomotor activity, as there was no major difference observed among groups in the total distances travelled in the open-field (Fig. 7C).

Next, we studied behavior in FCTs with respect to context- and cue-retention. Aβ_25–35_-injected mice demonstrated significantly less contextual- and cue-freezing responses compared with the sham mice (Fig. 7D,E), suggesting an impairment of associative memory. DAPK1 inhibitor administration significantly attenuated the impairment of contextual and cue freezing responses in Aβ_25–35_-injected mice.

## Discussion

In the present study, we showed that DAPK1 which was activated downstream of Aβ_25–35_-triggered lysosomal cathepsin B leakage, promoted caspase-1 activation and consequently IL-1β production in Bv2 cells and mice. Additionally, pharmacological inhibition of DAPK1 protected against memory deficits induced by the Aβ_25–35_ injection.

Aβ could activate microglia and initiate the release of various neurotoxic inflammatory cytokines and chemokines^22,27,28^. We found that Aβ_25–35_ which retains most of the neurotoxic properties of the full-length Aβ was able to induce robust IL-1β production and caspase-1 activation in LPS-primed cells in our current study^7,29^. This is consistent with a recent study which reported Aβ_25–35_ peptide triggered IL-1β secretion through activating the NLRP3 inflammasome in microglia^30^. Lysosomal protease cathepsin B has been implicated in the activation of the inflammasome induced by multiple particulate stimuli^31,32^. We here observed an increase in the lysosomal membrane permeabilization after stimulating LPS-primed Bv2 cells with Aβ_25–35_, which in turn caused the leakage of cathepsin B into the cytosol. This process seems to be responsible for the initiation of Aβ_25–35_-induced activation of the caspase-1/IL-1β pathway since we found that IL-1β secretion and caspase-1 activation were decreased in the presence of cathepsin B inhibitor.

Recent studies indicated a role of DAPK1 in several cellular processes and DAPK1 exerts its effects through an increase in its activity^33,34^. In the experimental setting here, LPS priming and Aβ_25–35_ stimulation led to a significant increase of DAPK1 activity in Bv2 cells, as evidenced by increased levels of p-MLC.

Prior studies revealed the involvement of DAPK in inflammatory responses. It has been demonstrated that DAPK1 negatively regulated the activation of the TNF-α and IFN-γ-stimulated NF-κB signaling^35^. However, DAPK promoted the assembly of the NLRP3 inflammasome in macrophages^18^. To explore a mechanistic insight into the role of DAPK1 in IL-1β production in microglia, we examined the effects of DAPK1 knockdown and overexpression, as well as DAPK1 catalytic activity inhibition on Aβ_25–35_-induced caspase-1 activation. We found that DAPK1 silencing and activity inhibition largely abolished Aβ_25–35_-induced IL-1β secretion and caspase-1 cleavage, while cDAPK1 attenuated these effects. We also observed that the expression of NLRP3, ASC, pro-caspase-1 and pro-IL-1β was not changed in the presence of DAPK1 knockdown or DAPK1 overexpression before and after the treatment of Aβ_25–35_ in LPS-primed Bv2 cells. DAPK1 knockdown and activity inhibition did not affect TNF-α and IL-6 release, as well as microglial viability. It appears that DAPK1 had little effect on the NF-κB-dependent priming stage of NLRP3 inflammasome activation. These results suggest that DAPK1 is a potent enhancer in the stage of caspase-1 activation, and are in keeping with a previous study in macrophages^18^.

In addition, DAPK1 was reported to be regulated by the lysosome. It is suggested that cathepsin B directly bind to DAPK1 in the TNFR-1-induced apoptosis, and that its deficiency increase the steady-state levels of DAPK1^26,36^. The region consisting of amino acids 836–947 in the DAPK1 was suggested to be essential for the binding of cathepsin B. Here, we observed a possibly telling connection between cathepsin B and DAPK1 by showing that cathepsin B inhibitor treatment dampened DAPK1 activation, while DAPK1 inhibitor had no effect on cathepsin B expression and release, which places cathepsin B upstream of DAPK1 activation. Our findings thus suggest a possibility that the cathepsin B-mediated increase in DAPK1 activation ensures that there are sufficient amounts of activated DAPK1 to participate in the activation of caspase-1 that will ultimately lead to IL-1β maturation.

Transfer of sensory information to the hippocampus mainly through the hippocampal trisynaptic circuitry (entorhinal cortex → dentate gyrus → CA3 → CA1 pathway)^37^. Despite the underlying neural circuitry supporting object recognition has not been clearly defined, there is some evidence so far to support the significant role of the hippocampus in the formation of objective memory^38–40^. ORT test session performance activated gene expression in the hippocampus (CA1 and CA3 sub-regions) and increased CA1 neurons firing rates^38,41^. More recent studies suggest that the amygdala and hippocampus operate in parallel during fear conditioning^42,43^. Hippocampal sub-regions CA1 and CA3 project to the amygdala via relays in the entorhinal cortex or through the ventroangular pathway directly^44^. A single intracerebroventricular (*i.c.v*.) administration of Aβ_25–35_ into the rodent brain provides a useful model to study neuroinflammation and behavior features^45^. NLRP3 inflammasome is primarily expressed in microglia in the brain^30,46^. Here, we observed increases in the Iba1 immunoreactivity and NLRP3 expression in the stratum radium of hippocampal CA1 and CA3 areas of Aβ_25–35_-injected mice, whereas there are no obvious changes in the pyramidal layer. Of particular importance, high levels of IL-1β have negative impacts on the processes of hippocampal long-term potentiation and synaptic plasticity^47–49^, which ultimately results in a decline in the cognitive performance. Consistent with the previous reports^46,50^, the *i.c.v*. injection of Aβ_25–35_ caused functional deficits in ORT and FCTs in our study. However, DAPK1 inhibitor significantly prevented Aβ_25–35_-evoked caspase-1 activation and IL-1β production, and rescued the decline in the object recognition and fear memory. Due to the fact that the inflammasome activation is closely associated with the neuronal injury^22,51^, we speculated that the neuroprotective effects of DAPK1 inhibitor, at least in part, may be related to its anti-inflammasome/caspase-1 activation effects.

Sufficient protein levels of NLRP3 are necessary to the formation and activation of the NLRP3 inflammasome. We observed an increase in NLRP3 expression *in vivo* and no alterations in LPS-primed Bv2 cells in response to Aβ_25–35._ Aβ injection results in a pro-inflammatory milieu in rodent brain and activation of the NF-κB signaling pathway^52^. In Bv2 cells, however, 6 h of LPS priming had robustly activated the expression of NLRP3 through the NF-κB pathway and following Aβ_25–35_ stimulation might mainly played a role in NLRP3 activation^18,31^.

Based on the findings of our present study, it is plausible that DAPK1 functions as an endogenous enhancer for Aβ_25–35_-induced IL-1β production through regulation of caspase-1 activation in microglia. Considering the crucial role of microglial inflammasome/caspase-1/IL-1β axis in neuroinflammation^8,53^, we propose that the inhibition of DAPK1 represents a potential therapeutic application for IL-1β-associated neurological diseases.

## Methods

### Peptide preparation

Aβ_25–35_ and Aβ_1–42_ (ChinaPeptides Co., Shanghai, China) were prepared as previouly described^22,54,55^. The Aβ_25–35_ was dissolved in sterile double-distilled water (3 mM) and incubated at 37 °C for 4 days to form “aged” Aβ_25–35_. To form Aβ_1–42_ fibrils, Aβ_1–42_ was dissolved in double-distilled water (10 mM) and incubated at 37 °C for 24 h. To prepare Aβ_1–42_ oligomers, Aβ_1–42_ was dissolved in dimethyl sulfoxide (DMSO, St. Louis, MO, USA) (5 mM) and incubated at 4 °C for 24 h. Aβ aggregates were diluted to the final concentration with phosphate buffer saline (PBS) just before each experiment.

### Cell culture and treatment

Bv2 cells, a murine microglial cell line, were obtained from Cell Resource Center of Peking Union Medical College (Beijing, China), and maintained in high-glucose DMEM supplemented with 10% FBS and 1% penicillin/streptomycin (all from Gibco, Grand Island, NY, USA) in a humidified incubator with 5% CO_2_ at 37 °C.

We primed cells with ultrapure lipopolysaccharide (LPS, 100 ng/ml) (Sigma-Aldrich, St. Louis, MO, USA) for 6 h and washed with PBS. After that, cells were further stimulated with Aβ_25–35_ (25 μM) or fibrillar Aβ_1–42_ (10 μM) or oligomeric Aβ_1–42_ (10 μM) for varying times (6–48 h). In some experiments, LPS-primed cells were incubated with cathepsin B inhibitor CA-074Me (1, 2.5, 5 μM; Calbiochem, Darmstadt, Germany) or DAPK1 inhibitor (2.5, 5, 10 μM; MedChem Express, Monmouth Junction, NJ, USA)) or for 1 h prior to Aβ_25–35_ treatment.

### DAPK1 knockdown and overexpression

To generate the stable DAPK1 knockdown cell line, lentiviral particles encoding DAPK1-specific shRNA or scrambled shRNA (Santa Cruz Biotechnology, CA, USA) were used to infect the Bv2 cells and polybrene (5 μg/ml) was added to the culture medium. After 6 h, the medium was replaced with DMEM containing 10% FBS. Two days later, puromycin (5 μg/ml) was added to the medium for selection. DAPK1 knockdown efficiency was confirmed by western blotting.

The DAPK1^ΔCaM^ mutant (here referred to as cDAPK1) which lacks a calcium/calmodulin regulatory domain was received as a gift from Prof. Lu (Institute for Brain Research, Department of Physiology and Key Laboratory of Neurological Diseases of Ministry of Education, Tongji Medical College, Huazhong University of Science and Technology, Wuhan, China) and was cloned into mammalian expression vector pRK5. DAPK1 knockdown cells or normal cells were transiently transfected with DAPK1 overexpression plasmids or empty plasmids using Lipofectamine 2000 (Invitrogen, Grand Island, NY, USA) according to the manufacturer’s protocols.

### Detection of cytokines and LDH

Cytokine concentrations in samples of culture supernatants and hippocampus homogenates were measured using the commercial ELISA kits (Boster Biological Technology, Wuhan, China) according to the manufacturer’s instructions.

The contents of LDH in samples of supernatants were quatitated by an LDH cytotoxicity assay (Nanjing Jiancheng, Nanjing, China) to evaluate the impact of different treatments on cell viability.

### Protein extraction and western blotting

Stimulated cells and tissue samples were harvested for protein extraction and western blotting analysis with standard protocols as previously described^12,56^. Cytoplasmic fractionations were performed with NE-PER Nuclear and Cytoplasmic Extraction Reagents (Thermo Scientific, Rockford, IL, USA) according to the manufacturer’ s protocol. Briefly, cells were harvested, washed and pelleted. Then added ice-cold CER I to the cell pellet and incubated the tube for 10 min on the ice. After that, added ice-cold CER II to the tube and incubated for 1 min on the ice. The volume ratios of cell pellets, CER I and CER II are 10: 100: 5.5. At last, centrifuged the tube for 5 min at 16, 000 × g and transferred the supernatant to a clean pre-chilled tube. Stored this tube in −80 °C until use. The following antibodies were used: anti-GAPDH (1:10000; Genetex, Irvine, CA, USA); anti-DAPK1, anti-MLC (1:1000; Abcam, Cambridge, MA, USA); anti-p-MLC (1:500; Cell Signaling Technology, MA, USA); anti-IL-1β (1: 2000; R&D Systems, Wiesbaden, Germany); anti-NLRP3, anti-caspase-1, anti-ASC (1:1000; Adipogen, San Diego, CA, USA)]; anti-cathepsin B (1:300; Santa Cruz Biotechnology, Santa Cruz, CA, USA); secondary antibodies (1:400; Jackson ImmunoResearch Laboratories, PA, USA). The intensity of protein bands was analyzed with the Image J software (NIH) and normalized to GAPDH.

### Determination of cathepsin B activity and the acidic compartment

Briefly, Bv2 cells were seeded on poly-L-lysine (Sigma-Aldrich, St. Louis, MO, USA) coated sterile coverslips, and treated as indicated. To monitor lysosome rupture and cathepsin B activity, treated Bv2 cells were incubated with acridine orange (AO) or with a fluorogenic cathepsin B substrate and Hoechst stain, according to the manufacturer’s instructions (CV-cathepsin B detection kit, BIOMOL International LP, Plymouth Meeting, PA, USA). The stained live cells were then observed using an Olympus immunofluorescence microscope (Olympus, Tokyo, Japan). The number of red staining cells was counted using Image-Pro Plus software (Rockville, MD, USA).

### Animals

Male C57BL/6 mice (8–10 weeks old) weighing 23–25 g were sourced from the experimental animal center of Wuhan University (Wuhan, China, No. 42000600003611) and maintained in a temperature and humidity controlled animal facility with ad libitum access to food and water and a 12 h light/ 12 h dark cycle. Adequate measures were made to minimize animals suffering during surgeries and reduce the number of animals used. Animal handling procedures in the present study were in accordance with the Guide for the Care and Use of Laboratory Animals of Tongji Medical College. All animal experiments were approved by the committee of experimental animals of Tongji Medical College of Huazhong University of Science and Technology.

### Preparation of animal model

Mice were anesthetized with intraperitoneal (*i.p*.) admininistration of 2% pentobarbitone sodium (0.06 ml/10 g) and placed in a stereotactic apparatus (RWD, Shenzhen, China). Body temperature was maintained at 37 °C using a heating pad. Aβ_25–35_ (3 nmol/mouse at 3 μl) was administered intracerebroventricularly (*i.c.v*.) using a microsyringe with a 28-gauge stainless-steel needle at a rate of 0.5 μl/min^57^. The injection site was confirmed by the injection of Evans blue dye (2%) in the preliminary experiments (see Supplementary Fig. S7). The mice *i.c.v*. injected with an equal volume of PBS served as the sham group. Before skull was exposed, the lidocaine cream was local administrated to each mouse to prevent pain.

For the acute treatment of DAPK1 inhibitor, mice were received a single *i.c.v*. injection of DAPK1 inhibitor (2.5 or 5 nmol/ mouse at 2 μl) 1 h before Aβ_25–35_ administration. Mice were divided into 5 groups: (I) sham + vehicle (n = 17), (II) sham + DAPK1 inhibitor (n = 12), (III) Aβ_25–35_ + vehicle (n = 17), (IV) Aβ_25–35_ + DAPK1 inhibitor (2.5 nmol) (n = 12), (V) Aβ_25–35_ + DAPK1 inhibitor (5 nmol) (n = 12). For the subchronic treatment of DAPK1 inhibitor, mice were injected *i.p*. with the DAPK1 inhibitor (1 mg/kg/day) for 8 consecutive days. Mice were divided into 4 groups (n = 11 per group): (I) sham + vehicle, (II) sham + DAPK1 inhibitor, (III) Aβ_25–35_ + vehicle, (IV) Aβ_25–35_ + DAPK1 inhibitor.

### Immunofluorescence analysis

Mice were anesthetized with pentobarbitone sodium (0.06 ml/10 g, *i.p*.) and perfused with 4% paraformaldehyde at 48 h after surgery. Brains were post-fixed in paraformaldehyde for 24 h, cryoprotected with 30% sucrose for 48 h, embeded into OCT compound (Torrance, CA, USA) and frozen at −80 °C overnight. Coronal sections (10 μm) including the hippocampus were prepared uisng a cryostat and mounted on precoated glass slides. Brain sections were blocked with 10% normal goat serum in PBS containing 0.3% Triton X-100 and incubated overnight at 4 °C with primary antibodies [NLRP3, 1:300; Iba1 (Wako, Japan), 1:300]. After washing, sections were incubated with IFKine Green AffiniPure Donkey Anti-Rabbit IgG and Dylight 549 Goat Anti-Mouse IgG (both from Abbkine, Redlands, CA, USA) (1:200) for 60 min at 37 °C and counterstained with DAPI (Roche, Mannheim, Germany) for 10 min. Subsequently, confocal images were acquired using a Nikon A1 confocal laser scanning microscope (Nikon, Japan). The numbers of Iba1-/NLRP3-double-positive cells were calculated in 10 coronal sections corresponding to the hippocampal CA1 and CA3 regions with the software Image-Pro Plus. Briefly, the images were analyzed by setting a threshold for all sections of a specific labeling. The stained area above the threshold was determined for each section. The co-localization of Iba1 and NLRP3 was then determined as the co-stained area and counted.

### Behavior testing

#### Open-field test

The open-field test (OFT) was used to measure the locomotor activity of the animals on day 7 after Aβ_25–35_ injection. A detailed description of this method is provided in Supplementary Methods.

#### Object recognition test

The object recognition test (ORT) was performed on the days 7–8 after Aβ_25–35_ injection in accordance with a standardized protocol described by Legar M and colleagues with some modifications^58^. A detailed description of this method is provided in Supplementary Methods.

#### Fear conditioning test

On the days 9–10 after peptide injection, fear conditioning tests (FCTs) were carried out as detailed in a previous report^46^. A detailed description of this method is provided in Supplementary Methods.

### Statistical analysis

All variance values were represented as the mean ± SEM. When assumptions of normality and equal variance were met, group comparisons were evaluated using unpaired two-tailed Student’s t-tests or one-way ANOVA followed by Bonferroni tests. Statistical analyses were performed using GraphPad Prism 6.0 (GraphPad Software Inc., San Diego, CA, USA). P < 0.05 was considered statistically significant.

## Electronic supplementary material

Supplementary Information
