# Supplementary material for: Death-associated protein kinase 1 mediates interleukin-1β production through regulating inlfammasome activation in Bv2 microglial cells and mice
Source: Sci Rep. 2018 Jul 2;8:9930. doi: 10.1038/s41598-018-27842-y (PMC6028446; doi:10.1038/s41598-018-27842-y)
Supplement: Supplementary file 1 — Supplementary Information [file 41598_2018_27842_MOESM1_ESM.doc]

**Death-associated protein kinase 1 mediates interleukin-1β** **production through regulating inlfammasome activation in Bv2 microglial cells and mice**

Limin Song^1, +^, Lei Pei^2, +^, Lisha Hu^1^, Shangwen Pan^4^, Wei Xiong^1^, Min Liu^1^, Yan Wu^3, **^, You Shang^4, *^, Shanglong Yao^1^

^1^ Department of Anesthesiology, Institute of Anesthesiology and Critical Care Medicine, Union Hospital, Tongji Medical College, Huazhong University of Science and Technology, Wuhan 430022, China

^2^ Department of Neurobiology, School of Basic Medicine, Tongji Medical Collge, Huazhong University of Science and Technology, Wuhan 430022, China

^3^ Department of Neurology, Union Hospital, Tongji Medical College, Huazhong University of Science and Technology, Wuhan 430022, China

^4^ Department of Critical Care Medicine, Institute of Anesthesiology and Critical Care Medicine, Union Hospital, Tongji Medical College, Huazhong University of Science and Technology, Wuhan 430022, China

^*^ Corresponding author. Department of Critical Care Medicine, Institute of Anesthesiology and Critical Care Medicine, Union Hospital, Tongji Medical College, Huazhong University of Science and Technology, Wuhan 430022, China.

E-mail address: [you_shang@yahoo.com](mailto:you_shang@yahoo.com) (Y. Shang)

^**^ Corresponding author. Department of Neurology, Union Hospital, Tongji Medical College, Huazhong University of Science and Technology, Wuhan 430022, China.

E-mail address: [wuyan_120@163.com](mailto:wuyan_120@163.com) (Y. Wu)

^+^ These authors contributed equally to this work.

**Supplementary Methods**

**Open-field test**

Mice were placed individually in an open field apparatus (50 cm × 50 cm × 40 cm, Tai Meng Technology Co., Ltd., Chengdu, China) and allowed to move freely. The total distance moved was recorded for a period of 5 min^1, 2^.

**Objetct recognition test**

The procedure of object recognition test (ORT) consists of the habituation, training and test session. Of note, the open-field test was used as the habituation session for ORT. The training session was on day 8 post Aβ_25-35_ administration. Mice were placed in the open-filed with two selfsame objects and the exploratory activity for 5 min was recorded. The test session was performed after a short intersession interval of 6 h. A novel object and a triplicate copy of the two familiar objects were placed in the same positions as in the training session. Mice were returned to the open-field and allowed to explore both objects for 5 min. After each experiment, the objects and the open-field were cleaned with 70% ethanol solution to minimize olfactory cues. Object exploration was defined as sniffing the object or touching the object with directing the nose toward it ^1, 3^.

The recognition index was calculated as the ratio of the time spent exploring the novel

object over the total time spent exploring two objects (T_new_) / (T_new_ + T_familiar_) ^4^.

**Fear conditioning test**

Before fear conditioning (FC) training, mice were habituated to the FC chamber (33 cm× 33 cm × 35cm, with steel grid bottom, Tai Meng Technology Co., Ltd., Chengdu, China), and allowed to explore for 2 min. For training, mice were exposed to three conditional stimuli cycles (30-s tone, 80 dB; 2-s electric footshock, 1 mA; 30-s interval). After 24 h, contextual- and cue-fear conditioning tasks were conducted. For the contextual-conditioning fear test, mice were re-exposed to the same chamber for 3 min without the footshock or tone. Cue-conditioning fear test was carried out 1 h later. Mice were placed in a different chamber for 2 min and then exposed to the same stimuli in the training phase for 3 min except the footshock. The “freezing” behavior was defined as the absence of any movement other than respiration. To assess memory, the percentage of time spent freezing were analyzed.

**References**

1. Souza, L.C., et al. Indoleamine-2,3-dioxygenase mediates neurobehavioral alterations induced by an intracerebroventricular injection of amyloid-β1-42 peptide in mice. Brain Behav immun. **56**, 363-377 (2016).

2. Prut, L., & Blezung, C. The open field as a paradigm to measure the effects of drugs on anxiety-like behaviors: a review. Eur J Phamacol. **463**, 3-33 (2003).

3. Leger, M., et al. Object recognition test in mice. Nat Protoc. **8**, 2531-2537 (2013).

4. Santos, D.B., et al. Probucol, a lipid-lowering drug, prevents cognitive and hippocampal synaptic impairments induced by amyloid β peptide in mice. Exp Neurol. **233**, 767-775 (2012).

**Supplementary Figures**

**
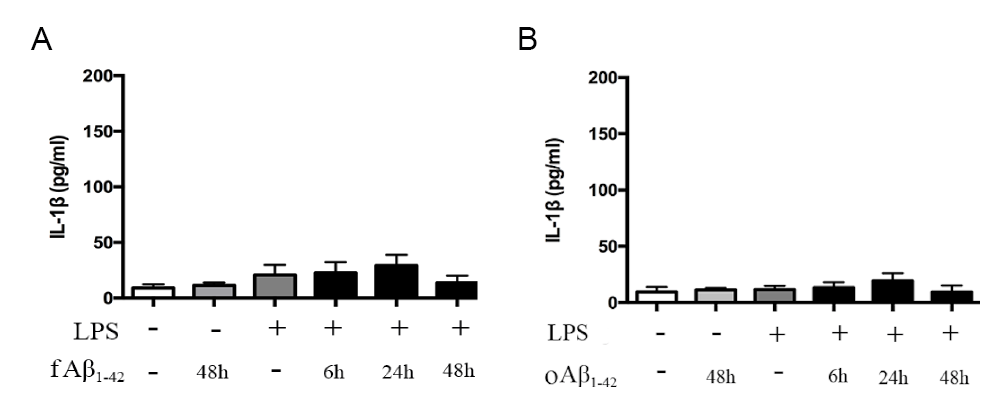
**

**Supplementary Figure S1**. β-amyloid (Aβ)_1-42_ failed to induce IL-1β secretion in LPS-primed Bv2 cells. Cells were primed with LPS (100 ng/ml) for 6 h, and treated with fibrillar Aβ_1-42_ (fAβ_1-42_, 10 μM) (A) or oligomeric Aβ_1-42_ (oAβ_1-42_,10 μM) (B) for varying times (6 h, 24 h, 48 h); the amount of IL-1β in the culture supernatant was assayed by ELISA.


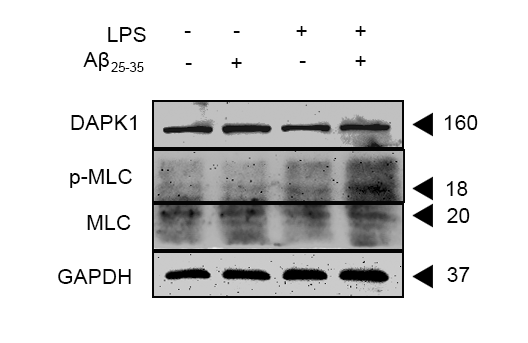


**Supplementary Figure S2**. Aβ_25-35_ induced DAPK1 activation in LPS-primed Bv2 cells. Cells were primed with LPS and stimulated with Aβ_25-35_ for 24 h. Protein levels of DAPK1 and p-MLC were assessed by western blotting analysis. Original blots are shown.


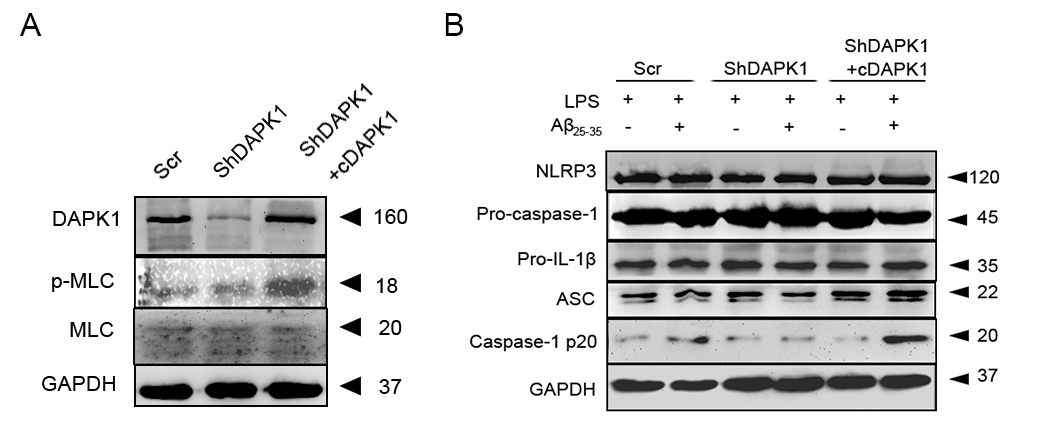


**Supplementary Figure S3**. DAPK1 is involved in Aβ_25-35_-induced caspase-1 activation in LPS-primed Bv2 cells. (A) Confirmation of DAPK1 knockdown and cDAPK1 expression in Bv2 cells by western blotting. (B) The effects of DAPK1 knockdown and cDAPK1 expression on the expression of caspase-1, pro-IL-1β, NLRP3 and ASC in LPS-primed, Aβ_25-35_-stimulated cells were determined by western blotting. Original blots are shown.


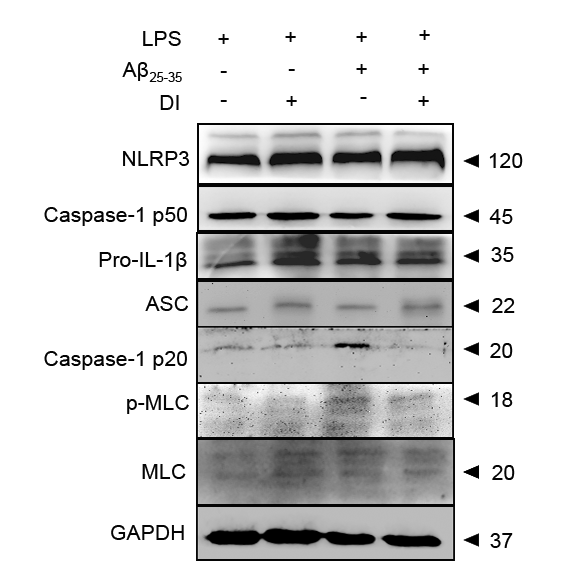


**Supplementary Figure S4**. Lack of DAPK1 activity impairs Aβ_25-35_-induced caspase-1 activation in LPS-primed Bv2 cells. The effects of DAPK1 inhibitor (10 μM) treatment on the expression of p-MLC, caspase-1, NLRP3, pro-IL-1β and ASC in LPS-primed, Aβ_25-35_- stimulated cells were determined by western blotting analysis. Original blots are shown. DI: DAPK1 inhibitor.


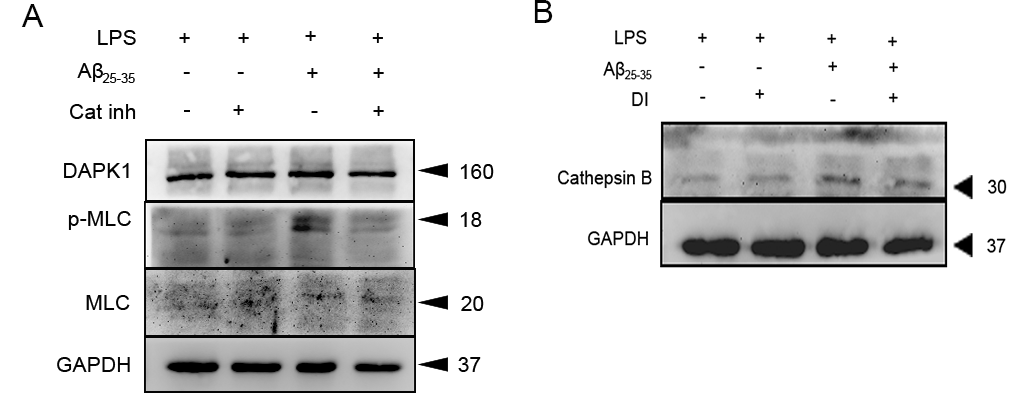


**Supplementary Figure S5**. Aβ_25-35_-induced cathepsin B acts upstream of DAPK1. (A) The effects of cathepsin B inhibitor (5 μM) treatment on the expression of DAPK1 and p-MLC in LPS-primed, Aβ_25-35_-stimulated Bv2 cells were analyzed by western blotting. (B) The effects of DAPK1 inhibitor (10 μM) treatment on the expression of cathepsin B in the cytoplasm in LPS-primed, Aβ_25-35_-stimulated Bv2 cells were analyzed by western blotting. Original blots are shown. Cat B inh: cathepsin B inhibitor.


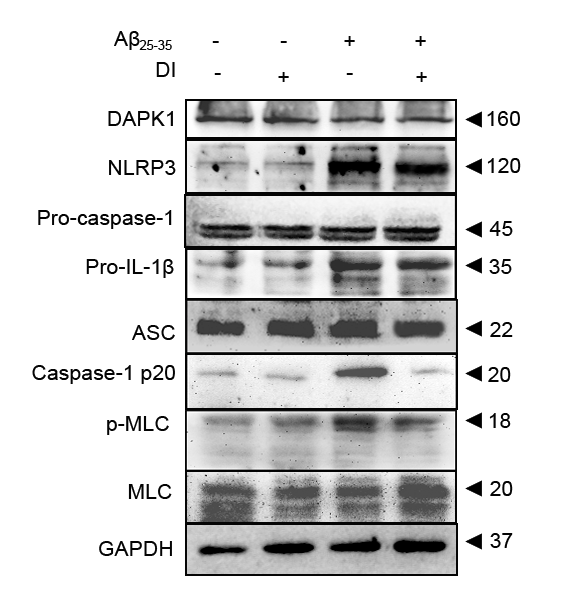


**Supplementary Figure S6**. Effects of DAPK1 inhibitor (5 nmol) treatment on Aβ_25-35_-induced NLRP3 inflammasome activation in the hippocampus of mice. The expression of DAPK1, p-MLC, NLRP3, caspase-1, pro-IL-1β and ASC in the hippocampus was determined by western blotting. Original blots are shown.


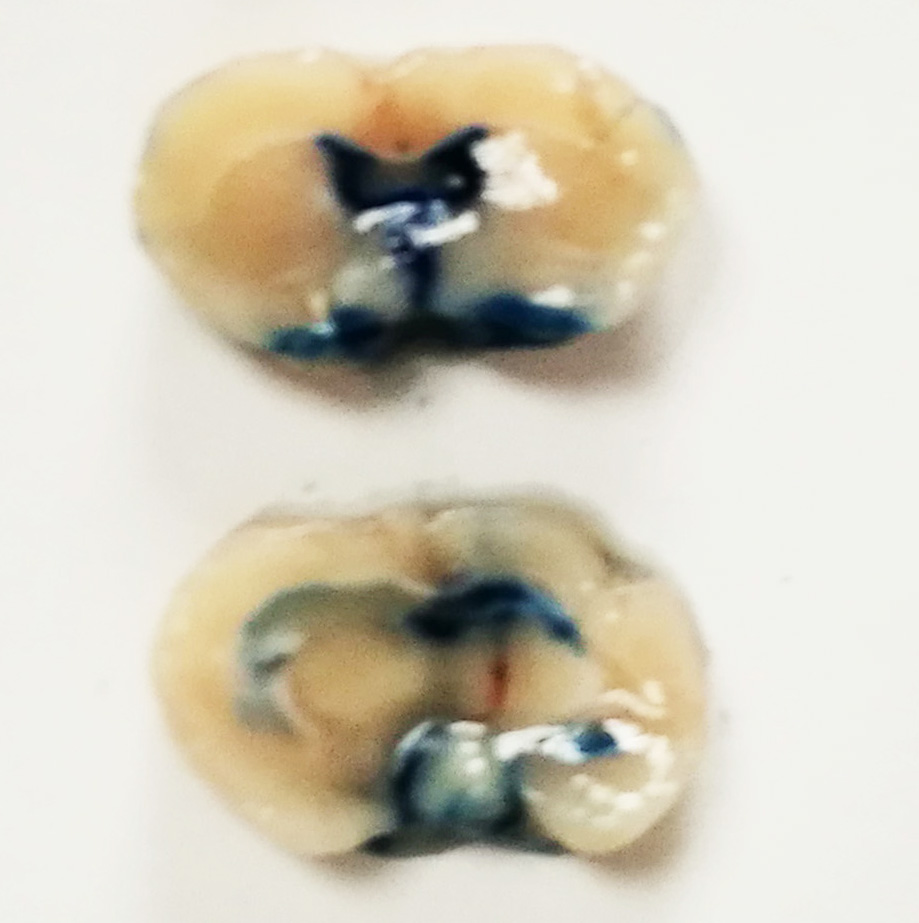


**Supplementary Figure S7.** The photomicrograph of the Evans blue dye to indicate the site of intracerebroventricular (*i.c.v.*) injection.
